# Testing the Role of Depression in the Relationship Between Socioeconomic Status and Cognitive Function Among Older Chinese Adults: Findings From the Anhui Healthy Longevity Survey

**DOI:** 10.31083/AP39349

**Published:** 2025-08-31

**Authors:** Chen Dai, Wenzheng Zhao, Danrui Yang, Guohan Fan, Qingzhou Meng, Hui Yang, Lunfang Xie, Yan Zhang, Xiaoli Zha

**Affiliations:** ^1^Department of Health Service Management, Anhui Medical University, 230032 Hefei, Anhui, China; ^2^Department of Nursing, The First Affiliated Hospital, Anhui Medical University, 230032 Hefei, Anhui, China

**Keywords:** cognitive function, depressive symptoms, mediation analysis, moderation analysis, older adults, socioeconomic status

## Abstract

**Objective:**

This study examined the relationships between socioeconomic status (SES), depression, and cognitive function in older adults, with a focus on the mediating or moderating role of depression in the link between SES and cognitive function.

**Methods:**

Data were analyzed from 5527 participants in the Anhui Healthy Longevity Survey (AHLS). SES was determined by educational attainment and individual annual income; depressive symptoms were assessed using the Patient Health Questionnaire-9 (PHQ-9); and cognitive function was evaluated with the Mini-Mental State Examination (MMSE). Linear regression analyses were conducted to investigate the relationships among SES, depressive symptoms, and cognitive function. The PROCESS macro in SPSS was utilized to perform both mediation and moderation analyses, following established procedures.

**Results:**

Compared to low SES, both medium SES (B = 4.115, *p* < 0.001) and high SES (B = 6.827, *p* < 0.001) were both positively associated with MMSE scores and negatively associated with PHQ-9 scores (B = –0.827, *p* < 0.001; and B = –1.695 –0.195, *p* < 0.001, respectively). Moreover, PHQ-9 scores were negatively associated with MMSE scores (B = –0.132, *p* < 0.001). Further analysis revealed that PHQ-9 scores partially mediated the relationship between SES and MMSE scores, with mediation effects accounting for 3.16% and 2.58% of the total effect in the high SES and medium SES groups, respectively. The absence of significant interaction between PHQ-9 scores and either high (B = 0.099, *p* = 0.109) or medium SES (B = 0.003, *p* = 0.919) suggests that depressive symptoms do not moderate the association between SES and cognitive function.

**Conclusion:**

Lower SES is associated with poorer cognitive performance, with depressive symptoms partially mediating the relationship between SES and cognition.

## Main Points

1. This study found that lower socioeconomic status (SES) was associated with poorer cognitive function in older adults.

2. Depressive symptoms showed a statistically significant partial mediation effect between SES and cognitive function, however the proportion mediated was modest.

3. These findings underscore the need to adopt comprehensive interventions that address both SES and mental health in order to improve cognitive performance in older adults.

## 1. Introduction

Cognitive impairments, including subtypes such as Alzheimer’s disease (AD) and other forms of dementia, are progressive neurodegenerative conditions commonly found in older adults [1]. With the increase in global life expectancy and growth of the aging population, the prevalence of cognitive impairment among older adults is rising [2]. In China, the overall prevalence of dementia among individuals aged 60 years and older is 6.0%, corresponding to approximately 15.07 million people. AD accounts for the majority of these cases, with an estimated 9.83 million patients. Additionally, the early stage of dementia—mild cognitive impairment (MCI)—has a notably high prevalence of 15.5% in the same age group, affecting an estimated 38.77 million individuals [3].

Cognitive impairment significantly hinders social interactions and overall well-being, frequently resulting in premature mortality and contributing substantially to the burden of disease [4]. It can severely affect an individual’s ability to perform daily tasks, leading to a loss of independence and increased reliance on caregivers [5]. As cognitive decline progresses, it often impairs communication, decision-making, and memory, creating challenges for both the sufferers and their families [6]. In addition to the personal toll, cognitive decline also imposes a significant economic burden on society, including costs associated with care, hospitalization, and support services. The psychological and emotional effects on both patients and caregivers can be profound, potentially leading to depression, anxiety, and heightened stress [7].

Despite these challenges, current pharmacological treatments for cognitive impairment remain limited [8]. Consequently, research has increasingly focused on the identification of modifiable risk factors that could delay cognitive decline. Numerous studies have highlighted the role of chronic conditions [9], unhealthy lifestyles [10, 11], genetic inheritance [12, 13], and psychological factors [14, 15]. Among these, social risk factors, particularly socioeconomic status (SES) [16], have been found to influence cognitive function in later life. Early educational attainment and income levels during adulthood have been consistently associated with better cognitive function in older adults [17]. Although most studies have reported a positive association between higher SES (typically characterized by higher education and income levels) and higher levels of cognitive health [18, 19], some evidence has also indicated that individuals with high SES may experience a faster decline in cognitive function in later years due to age-related factors [20].

Depression is another critical public health concern among older adults. According to a recently published meta-analysis, the overall prevalence of depressive symptoms in the older population was estimated at 20.0% [21], highlighting the growing importance of preventing and managing depression in China’s aging population. Many studies have demonstrated a significant association between depressive symptoms and cognitive performance. For instance, a meta-analysis showed that individuals with a history of depression had twice the risk of developing dementia [22], suggesting that depressive symptoms may play a role in the onset of cognitive impairment [23]. Moreover, depression, in conjunction with SES, also exerts an influence on cognitive health [24]. As a potential moderator, depressive symptoms may diminish the protective effects of high SES on cognitive performance. For example, individuals with depressive symptoms might not fully utilize the cognitive advantages associated with high SES [25]. Additionally, depression may act as a mediator in this association. Lower SES often exposes individuals to resource constraints and chronic stressors, thus increasing their vulnerability to depressive symptoms [26, 27] which in turn can negatively affect their cognitive health [28]. This dual role of depression underscores the complexity of its influence on the SES-cognition association.

Understanding whether depressive symptoms influence the link between SES and cognitive function is essential for developing effective interventions to enhance cognitive resilience in older adults. However, research on the moderating or mediating roles of depressive symptoms in this context has so far been limited. In the current study, we investigated whether, and how, depressive symptoms affect the SES-cognition relationship among older adults in Anhui, China. Specifically, the following hypotheses (H) were tested:

“Ha” represents mediating effect hypotheses: Ha1: SES has a significant direct effect on cognition. Ha2: SES has a significant effect on depressive symptoms. Ha3: Depressive symptoms have a significant effect on cognition. Ha4: Depressive symptoms partially mediate the association between SES and cognition. Ha5: Depressive symptoms fully mediate the association between SES and cognition.

“Hb” represents moderating effect hypotheses: Hb1: Depressive symptoms significantly moderate the relationship between SES and cognition. Hb2: The interaction between SES and depressive symptoms has a significant effect on cognition.

## 2. Methods

### 2.1 Study Design and Data Collection

Participants in the current study were selected from the Anhui Healthy Longevity Survey (AHLS), as detailed in previous reports [29]. We used a multistage sampling approach across four geographically representative cities in Anhui Province: Lu’an (western), Xuancheng (southern), Chuzhou (eastern), and Fuyang (northwestern). Within each city, 3–5 communities were selected from both urban and rural areas based on three criteria: economic status, geographic distribution, and logistical feasibility. This strategy ensured socioeconomic diversity while maintaining field-research practicality. No sampling weights were applied in this study, as it utilized a purposive sampling approach. Individuals aged ≥60 years from these areas and who possessed basic communication skills were encouraged to take part.

Participants completed structured questionnaires to provide data on their demographic details, health behaviors, and chronic illnesses. Prior to the study, all data collectors underwent standardized training to ensure consistency and accuracy in the data-collection process. On the survey days, the completed questionnaires were cross-checked for quality, and any incomplete or unclear responses were verified and corrected. To further ensure data accuracy, a double-entry process was implemented, and any discrepancies were thoroughly reviewed and resolved.

### 2.2 SES Classification

SES was assessed based on the education level of participants and their self-reported individual annual income (i.e., personal income over the past year, excluding income from other household members). Although occupation is also a commonly used indicator for assessing SES, it was not included in this study, as most individuals in China who are aged 60 years and above are retired. Education level was categorized into three groups: low (illiterate), medium (1–6 years), and high (>6 years). This classification reflects the generally low educational attainment among older adults in China, with almost half the participants in this study classified as illiterate. Individual annual income was categorized into two groups: low (<6500 yuan ≈ 940 USD) and high (≥6500 yuan), based on the estimated median individual income level for the study population. The overall SES of participants was classified into three categories according to the combination of education and individual income levels: low SES (low education and income), medium SES (various combinations of education and income levels, such as higher income with lower education, or lower income with medium or higher education), and high SES (high education and income) [19]. By integrating both the education level and individual annual income into the SES assessment, we sought to mitigate potential biases that may have arisen from relying solely on education. This composite measure of SES offers a more comprehensive and balanced evaluation of the SES of each participant.

### 2.3 Definition of Depressive Symptoms

Depressive symptoms for each participant were assessed using the Patient Health Questionnaire-9 (PHQ-9), a self-report scale with 9 items for measuring depressive symptoms. Scores on the PHQ-9 range from 0 to 27, with higher scores indicating a greater severity of depression [30]. In the present study, the PHQ-9 demonstrated strong internal consistency, with a Cronbach’s α of 0.831, thereby confirming its reliability [31].

### 2.4 Evaluation of Cognitive Function

The Mini-Mental-State Examination (MMSE) was used to evaluate cognitive function. This widely recognized tool is known for its accuracy and reliability in assessing cognitive skills [32]. The MMSE assesses various cognitive domains, including orientation, registration, attention, calculation, recall, and language. The maximum score is 30 points, with higher scores indicating better cognitive performance. In the present study, the MMSE demonstrated satisfactory internal consistency, with a Cronbach’s α of 0.725.

### 2.5 Assessment of Covariates

Various demographic characteristics and potential confounding factors were included as covariates in this study, included age (continuous), gender (male, female), residential region (urban, rural), marital status, living arrangement (living alone or not), drinking status (current drinker or not), smoking status (current smoker or not), sedentary duration, and chronic conditions such as hypertension, hyperlipidemia, and diabetes. Marital status was categorized into married or unmarried (including divorced, widowed, or never married). Living arrangement was assessed by asking the participant: “Do you live alone?”, with responses categorized as “yes” or “no”. Drinking status was categorized into current drinkers and non-current drinkers. Similarly, smoking status was divided into current smokers and non-smokers. Participants reported their physical activity levels by indicating their daily sedentary time, which was categorized into three groups: <3 h, 3–5 h, and >5 h. Additionally, chronic illnesses, including diabetes, hypertension, and hyperlipidemia were assessed based on whether the participant had received a diagnosis from medical professionals at the county level or higher. Responses were recorded as “yes” or “no”.

### 2.6 Statistical Analyses

The mean and standard deviation were used to describe continuous variables, while percentages were used for categorical variables. Harman’s single-factor test was applied to assess common method bias using unrotated principal component analysis. In this test, the presence of common method bias is indicated if a single factor emerges, or if the first principal factor accounts for >40% of the total variance. The analysis identified several factors with eigenvalues >1, while the primary principal factor accounted for <40% of the variance, indicating the absence of significant common method bias [33].

Linear regression analyses were conducted to examine pairwise associations among SES, depressive symptoms, and cognitive function. Two analytical models were used. The first was an unadjusted linear regression model, while the second was an adjusted linear regression model that included the following covariates: age, gender, residential area, current smoking status, current alcohol consumption status, marital status, living arrangement, diabetes, hypertension, hyperlipidemia, and sedentary time.

Prior to conducting mediation and moderation analyses, key assumptions such as linearity, normality, and multicollinearity were examined, with the results confirming their acceptability. Mediation and moderation analyses were performed using the PROCESS macro in SPSS 26.0 (IBM Corp., Armonk, NY, USA), with maximum likelihood estimation to assess the hypothesis. To assess indirect effects, 95% bias-corrected confidence intervals (CIs) were calculated using a bootstrapping method with 5000 resamples. The bootstrap method was selected because it does not require the assumption of normality for the sampling distribution of the indirect effect, and is thus considered a robust approach to estimating mediation effects [34]. Given that SES is a trinomial variable, an omnibus-mediating-effect analysis was initially conducted [35]. The present study examined whether depressive symptoms acted as mediators in the relationship between SES and cognitive function. For the moderation analysis, the PROCESS macro was used to assess whether depressive symptoms moderated the relationship between SES and cognitive function. Both the mediation and moderation models were adjusted for the same covariates as the regression models.

Multivariable linear regression models were also conducted to examine the associations between SES and depressive symptoms, with cognitive function as a sensitivity analysis after adjustment for the covariates. This analysis was used to verify the robustness of the main findings derived from the mediation and moderation models using the bootstrap method.

Statistical analyses were conducted using SPSS 26.0 software. Two-tailed tests were performed, with statistical significance set at *p* ≤ 0.05.

## 3. Results

**Fig. 1.**
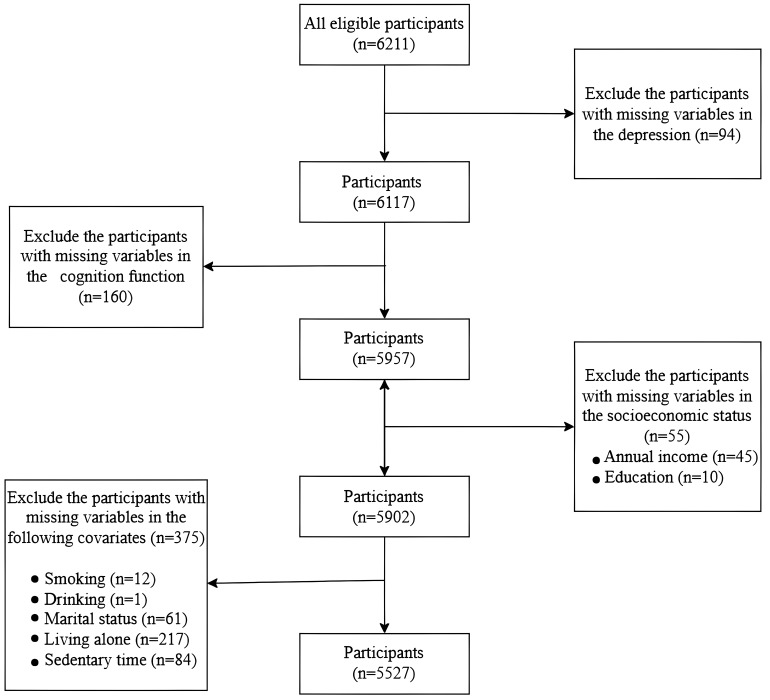
**Flow chart for participant selection**.

A total of 6211 participants, aged 60 years and older were recruited between July 2019 and August 2019. The flowchart detailing the participant selection process for the analytic sample is shown in Fig. 1. Participants with missing data for depressive symptoms (*n* = 94), SES (*n* = 55), cognitive function assessment (*n* = 160), or covariates (*n* = 375) were excluded. After these exclusions, the final analytic sample comprised 5527 participants.

The participant details are presented in Table 1. The 5527 participants comprised 2520 men and 3007 women, with a mean age of 71.03 years. Nearly half the participants (49.7%) were illiterate, and the majority (72.2%) were married. Over 60% reported individual annual incomes of <6500 yuan, while the mean MMSE score was 21.51.

**Table 1.** **Basic demographics of participants**.

		n	Mean (SD)/Percentage
Age (years)		5527	71.03 (7.10) *
Gender			
	Male	2520	45.6
	Female	3007	54.4
Residential region			
	Urban	2717	49.2
	Rural	2810	50.8
Education level			
	Low (0 years of education)	2746	49.7
	Medium (1–6 years of education)	1525	27.6
	High (>6 years of education)	1256	22.7
Marital status			
	Married	3988	72.2
	Other	1539	27.8
Living alone			
	Yes	1030	18.6
Current smoker			
	Yes	1176	21.3
Current drinker			
	Yes	2148	38.9
Diabetes			
	Yes	861	15.6
Hypertension			
	Yes	2779	50.3
Hyperlipidemia			
	Yes	409	7.4
Sedentary Duration (h/day)			
	<3	1484	26.9
	3–5	2508	45.4
	>5	1535	27.8
Individual annual income (yuan)			
	<6500 ^a^	3362	60.8
	≥6500 ^a^	2165	39.2
PHQ-9 score		5527	3.73 (4.35) *
MMSE score		5527	21.51 (6.13) *
Socioeconomic status			
	Low	2069	37.4
	Medium	2578	46.6
	High	880	15.9

* Data are expressed as the mean (SD). PHQ-9, Patient Health Questionnaire-9; MMSE, Mini Mental State Examination. ^a^ 6500 yuan ≈ 940 USD.

Exploratory factor analysis revealed that five factors had eigenvalues >1, with the first factor accounting for 21.07% of the variance. This suggested minimal common method bias.

The initial analysis tested the association between SES and MMSE scores (**Supplementary Table 1**). Participants with medium SES had higher cognitive scores than those with low SES scores (B = 4.224, *p* < 0.001). In the second step, PHQ-9 scores were used as the dependent variable, with SES as the independent variable (**Supplementary Table 2**; B = –0.827, *p* < 0.001). In the third step, PHQ-9 scores were used as the independent variable, and MMSE scores as the dependent variable, revealing a significant negative association (B = –0.194, *p* < 0.001; **Supplementary Table 3**). These results were consistent for participants with high SES, indicating that a mediation analysis was appropriate. The findings also support the validity of hypotheses Ha1-Ha3.

Given the categorical nature of SES, an omnibus mediation analysis was conducted prior to the relative mediation analysis. The results showed that the relative total effects and the direct effects were both significant, as indicated by the omnibus total- and direct-effects tests (F = 517.66, *p* < 0.001 and F = 551.06, *p* < 0.001, respectively). Additionally, the bootstrap CIs for the omnibus-mediating-effect test did not include zero (–0.003, –0.001), confirming the presence of mediation effects.

**Fig. 2.**
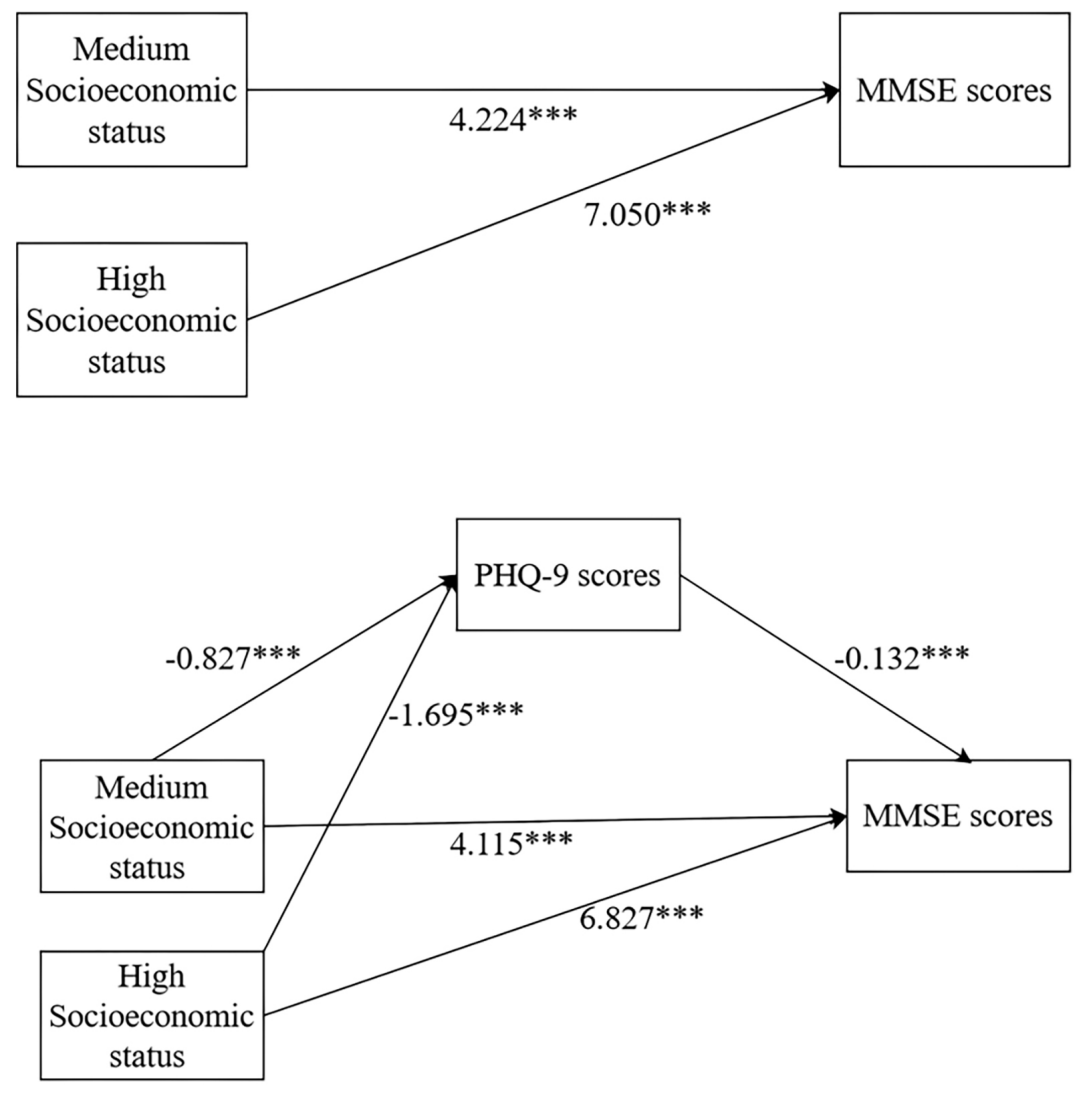
Visual illustration of PHQ-9 scores as a partial mediator for the association between SES and MMSE scores (***p < 0.001).

The regression analysis presented in Table 2 assessed the relationship between SES and MMSE scores, while Table 3 shows the mediation effects of PHQ-9 scores for medium SES (as illustrated in Fig. 2). The indirect effect was 0.109 (95% CI: 0.067–7.499), while the direct effect was 4.115 (95% CI: 3.804–4.426). In both cases, the 95% CI excluded zero, indicating that PHQ-9 scores partially mediated the relationship between medium SES and MMSE scores. The total effect, which is the sum of the direct and indirect effects, was 4.224. Thus, the direct effect of medium SES accounted for 97.42% of the total effect, while the indirect effect accounted for 2.58%. Similarly, for high SES, PHQ-9 scores partially mediated the association with MMSE scores, with the direct effect contributing 96.84%, and the indirect effect contributing 3.16%. These findings support hypothesis Ha4, whereas hypothesis Ha5 is not supported.

**Table 2.** **Bootstrap test of SES, PHQ-9 scores and MMSE scores**.

Regression model		Model fitting			Coefficient significance		
Outcome Variable	Independent Variable	R	R^2^	F	B (95% CI)	T	*p*
	Low SES (reference)						
MMSE scores	Medium SES	0.582	0.339	201.97	4.115 (3.804, 4.425)	25.970	<0.001
	High SES				6.827 (6.372, 7.281)	29.450	<0.001
	PHQ-9 scores				–0.132 (–0.163, 0.099)	–8.145	<0.001
	Low SES (reference)						
PHQ-9 scores	Medium SES	0.292	0.085	39.659	–0.827 (–1.085, –0.569)	–6.286	<0.001
	High SES				–1.695 (–2.071, –1.319)	–8.833	<0.001

The model was adjusted for age, gender, residential region, current smoker, current drinker, marital status, living alone, diabetes, hypertension, hyperlipidemia and sedentary duration. SES, Socioeconomic Status.

**Table 3.** **The mediating effect of PHQ-9 scores on SES and MMSE scores (partial mediation)**.

Path		Effect size	Boots SE	LLCI	ULCI	Proportion mediated
Total effect^a^		4.224	0.159	3.912	0.158	
	Indirect effect	0.109	0.023	0.067	7.499	2.58%
	Direct effect	4.115	0.159	3.804	4.426	97.42%
Total effect^b^		7.050	0.231	6.592	7.501	
	Indirect effect	0.223	0.038	0.155	0.301	3.16%
	Direct effect	6.827	0.232	6.372	7.281	96.84%

^a^ Medium Socioeconomic status →MMSE scores. ^b^ High Socioeconomic status →MMSE scores. The model was adjusted for age, gender, residential region, current smoker, current drinker, marital status, living alone, diabetes, hypertension, hyperlipidemia and sedentary duration. Boots SE: Bootstrap standard error; LLCI, lower limit of confidence interval; ULCI, upper limit of confidence interval.

The results of moderation analysis presented in Table 4 indicated the interaction between medium SES and PHQ-9 scores did not significantly affect MMSE scores (B = 0.003, *p* = 0.919). Similarly, the interaction effect between high SES and PHQ-9 scores was not significant (B = 0.099, *p* = 0.109). These findings suggest that PHQ-9 scores do not influence the relationship between SES and cognitive function. Therefore, hypotheses Hb1 and Hb2 are not supported.

**Table 4.** **The moderating effect of PHQ-9 scores on SES and MMSE scores**.

Regression model		Model fitting			Coefficient significance		
Outcome Variable	Independent Variable	R	R^2^	F	B (95% CI)	T	*p*
	Low SES (reference)						
MMSE scores	Medium SES				4.080 (3.671, 4.489)	19.560	<0.001
	High SES				6.587 (6.034, 7.141)	23.360	<0.001
	PHQ-9 scores	0.584	0.341	167.320	–0.139 (–0.183, 0.094)	–6.051	<0.001
	Medium SES × PHQ-9 scores				0.003 (–1.061, 0.068)	0.102	0.919
	High SES × PHQ-9 scores				0.099 (–0.022, 0.220)	1.604	0.109

The model was adjusted for age, gender, residential region, current smoker, current drinker, marital status, living alone, diabetes, hypertension, hyperlipidemia and sedentary duration.

As shown in **Supplementary Table 4**, the sensitivity analysis using multivariable linear regression showed similar results to those of the main analysis. Specifically, medium and high SES were positively associated with MMSE scores (medium SES: B = 4.12, 95% CI: 3.80–4.43, *p* < 0.001; high SES: B = 6.83, 95% CI: 6.37–7.28, *p* < 0.001), whereas higher PHQ-9 scores were negatively associated with cognitive function (B = –0.13, 95% CI: –0.16 – –0.10, *p* < 0.001).

## 4. Discussion

The present study identified depression as a key mediator in the association between SES and cognitive function. Individuals with lower SES are more susceptible to depression, which in turn negatively affects their cognitive capacity. However, depression did not significantly moderate this relationship. This finding highlights the complexity of the interplay between SES, mental health, and cognitive abilities. Moreover, it suggests that depression primarily acts as a pathway through which SES affects cognitive function, rather than altering the strength of this association. The current study is one of the first to investigate the simultaneous relationships between SES, depression, and cognitive function in community-dwelling, older adults in Anhui, China. These results highlight the need to consider psychological health when examining the association between SES and cognitive function.

Previous study has generally reported a positive association between higher SES and better cognitive outcomes, with many finding that individuals with higher education and income levels have higher cognitive function than those with lower SES [18]. However, most of these studies did not account for potential mediators or moderators of SES-cognitive relationships [36, 37]. Previous research also showed a gradient relationship between SES and depression [38], which in turn is associated with poorer cognitive performance [39]. Our study contributes to the existing literature by demonstrating that depression mediates the relationship between SES and cognitive function. This finding highlights the significant influence of SES on cognitive health and underscores the importance of developing interventions that target depression as a modifiable factor.

Several mechanisms may explain how depression mediates the relationship between SES and cognitive function. Individuals with lower SES often experience heightened levels of social and biological stress. These stressors include increased financial strain [25], limited access to high-quality health services [40], and structural brain changes associated with emotional regulation, such as alterations in surface area and Intracranial Volume (ICV) [41]. Depression negatively affects cognitive function through mechanisms that include impaired memory, attention, and executive functions [42]. Depressive symptoms may initiate a glucocorticoid cascade that negatively affects the hippocampus, a crucial brain region for memory formation and storage [43]. Depression can also restrict social interaction, thereby diminishing the effects of support networks [44, 45] and increasing the risk of cognitive decline in older adults.

Despite its significance, the mediating effect of depression was found to be relatively small in the present study, suggesting that additional, unidentified factors may be influencing the link between SES and cognitive function. To better understand these relationships, future studies should investigate other potential mediators, including resource access, lifestyle behaviors, and various social determinants of health.

The absence of a moderating effect of depression may be attributable to several factors. First, unmeasured factors not accounted for in this study could influence the relationship between SES and cognitive function. Second, SES may affect cognitive function through biological pathways that are independent of depression, such as changes in brain structures related to cognitive function [46]. Third, the association between depressive symptoms and cognitive function might be non-linear, with notable effects potentially emerging only after surpassing a specific depressive severity threshold [47]. A non-linear relationship could potentially limit the role of depression as a moderator.

By employing mediation and moderation analyses, the current study comprehensively examined the relationships among SES, depressive symptoms, and cognitive function. This analytical approach elucidated underlying psychological mechanisms, thereby providing theoretical support for targeted interventions in related fields. However, the current study also had several limitations. First, its cross-sectional design limited our ability to establish causality and to fully ascertain the temporal order among SES, depression, and cognitive function. Longitudinal studies are thus needed to address this limitation. Despite the cross-sectional nature of the study, it is important to note that educational attainment occurs early in life and is usually established before the onset of depression and cognitive decline. Therefore, although our study was cross-sectional, the temporal precedence of education lends some support to the plausibility of the proposed mediation pathway. Second, the reliance on self-reported income and education for SES classification may introduce potential measurement bias. Although these two indicators are commonly used in large-scale epidemiological studies due to their practicality and availability, future studies may consider using objective measures of SES, such as income tax records, to improve the accuracy of SES classification. Third, although our study included a substantial sample of more than 6000 older adults from four geographically diverse cities in Anhui Province (representing the southeast, south, west, and north regions), the purposive sampling method may have limited the generalizability of our findings to the broader older population in China. Future multi-center studies using probability sampling designs would help to verify and extend these findings to more representative populations. Fourth, although both the PHQ-9 and MMSE are widely used tools with validated specificity and sensitivity, their inherent limitations warrant caution in their application. The PHQ-9 is not the diagnostic “gold standard” for clinical depression and may oversimplify the depressive symptoms. Similarly, the MMSE may not adequately capture cognitive function, particularly because it is significantly influenced by the participants’ educational background. The modest mediation-effect size observed in this study suggests that other unmeasured factors, such as social support and genetic predisposition, are likely to play significant roles in the relationship between SES, depression, and cognitive function. Therefore, future research should prioritize the use of more comprehensive diagnostic assessments and objective measures for the studied variables.

## 5. Conclusion

The present study investigated the factors linking SES, depression, and cognitive function among community-dwelling older adults in China. The results underscore the importance of mental health in alleviating the negative impact of low SES on cognitive health. Furthermore, our findings indicate that depression partially mediates the relationship between SES and cognitive function, but without a moderating effect. Although the mediating effect of depression was statistically significant, it was relatively modest, suggesting that other potential mediators may also contribute to this relationship. These findings highlight the need to adopt comprehensive preventive measures for improving cognitive health that include not only SES, but also other influencing factors. Our study provides a foundation and new perspectives to explore additional mediators and moderators that could further elucidate the complexity of these associations. Interventions that target depression remain promising strategies for enhancing cognitive resilience among socioeconomically disadvantaged, older adults. However, broader and multifaceted approaches are needed for optimal outcomes.

## Availability of Data and Materials

The original contributions presented in the study are included in the article, further inquiries can be directed to the corresponding authors.

## Supplementary Material

Supplementary material associated with this article can be found, in the online version, at https://doi.org/10.31083/AP39349.
