# Supplementary material for: Testing the Role of Depression in the Relationship Between Socioeconomic Status and Cognitive Function Among Older Chinese Adults: Findings From the Anhui Healthy Longevity Survey
Source: Alpha Psychiatry. 2025 Aug 31;26(5):39349. doi: 10.31083/AP39349 (PMC12593830; doi:10.31083/AP39349)
Supplement: Supplementary file 1 [file 2757-8038-26-5-39349-s1.docx]

**Supplementary Table 1. Association Between SES and MMSE Scores**

|  |  | **Unadjusted** **linear regression** | | | **Adjusted linear regression**  **controlling for covariates** | | | |
| --- | --- | --- | --- | --- | --- | --- | --- | --- |
|  |  | ***B(95%CI)*** | ***T*** | ***p*** |  | ***B(95%CI)*** | ***T*** | ***p*** |
| **Socioeconomic status** |  |  |  |  |  |  |  |  |
| Low (ref) |  |  |  |  |  |  |  |  |
| Medium |  | 5.187 (4.885, 5.490) | 33.614 | <0.001 |  | 4.224 (3.912, 4.535) | 26.593 | <0.001 |
| High |  | 8.807 (8.395, 9.220) | 41.858 | <0.001 |  | 7.050 (6.596, 7.504) | 30.444 | <0.001 |

Outcome variable: MMSE scores

Covariates: age, gender, residential region, current smoker, current drinker, marital status, living alone, diabetes, hypertension, hyperlipidemia and sedentary duration.

**Supplementary Table 2. Association Between SES and PHQ-9 Scores**

|  |  | **Unadjusted** **linear regression** | | |  | **Adjusted linear regression**  **controlling for covariates** | | |
| --- | --- | --- | --- | --- | --- | --- | --- | --- |
|  |  | ***B(95%CI)*** | ***T*** | ***P*** |  | ***B(95%CI)*** | ***T*** | ***p*** |
| **Socioeconomic status** |  |  |  |  |  |  |  |  |
| Low (ref) |  |  |  |  |  |  |  |  |
| Medium |  | -1.367 (-1.613, -1.121) | -10.909 | <0.001 |  | -0.827 (-1.085, -0.569) | -6.286 | <0.001 |
| High |  | -2.680 (-3.015, -2.345) | -15.684 | <0.001 |  | -1.695 (-2.076, -1.319) | -8.833 | <0.001 |

Outcome variable: PHQ-9 scores

Covariates: age, gender, residential region, current smoker, current drinker, marital status, living alone, diabetes, hypertension, hyperlipidemia and sedentary duration.

**Supplementary Table 3. Association Between PHQ-9 Scores and MMSE Scores**

|  |  | **Unadjusted** **linear regression** | | |  | **Adjusted linear regression**  **controlling for covariates** | | |
| --- | --- | --- | --- | --- | --- | --- | --- | --- |
|  |  | ***B*** | ***T*** | ***p*** |  | ***B*** | ***T*** | ***p*** |
| PHQ-9 scores |  | -0.326 (-0.362, -0.289) | -17.632 | <0.001 |  | -0.194 (-0.228,0.160) | -11.096 | <0.001 |

Outcome variable: MMSE scores

Covariates: age, gender, residential region, current smoker, current drinker, marital status, living alone, diabetes, hypertension, hyperlipidemia and sedentary duration.

**Supplementary Table 4. Multiple Linear Regression Analysis of MMSE Scores**

| ***Variables*** | ***B (95% CI)*** | ***T*** | ***p*** |
| --- | --- | --- | --- |
| Low SES (ref) |  |  |  |
| Medium SES | 4.115 (3.804, 4.425) | 25.97 | <0.001 |
| High SES | 6.827 (6.372, 7.281) | 29.45 | <0.001 |
| PHQ-9 scores | -0.132 (-0.163, -0.099) | -8.145 | <0.001 |

Outcome variable: MMSE scores

The model was adjusted for age, gender, residential region, current smoker, current drinker, marital status, living alone, diabetes, hypertension, hyperlipidemia and sedentary duration.
